# Supplementary material for: A Cruciform Petal-like (ZIF-8) with Bactericidal Activity against Foodborne Gram-Positive Bacteria for Antibacterial Food Packaging
Source: Int J Mol Sci. 2022 Jul 6;23(14):7510. doi: 10.3390/ijms23147510 (PMC9318148; doi:10.3390/ijms23147510)
Supplement: Supplementary file 1 [file ijms-23-07510-s001.zip › ijms-1758602-supplementary.pdf]

A cruciform petal-like (ZIF-8) with bactericidal activity against foodborne Gram-positive bacteria for antibacterial food packaging

Bowen Shen[1], Yuxian Wang[1], Xinlong Wang[2], Fatima Ezzahra Amal[2], Liying Zhu[3,*], Ling Jiang[1, 2,*]

[1]College of Biotechnology and Pharmaceutical Engineering, State Key Laboratory of Materials-Oriented Chemical Engineering, Nanjing 211816, China;

[2]College of Food Science and Light Industry, Nanjing Tech University, Nanjing 210009, PR China;

[3]School of Chemistry and Molecular Engineering, Nanjing Tech University, Nanjing 210009, PR China.

[*]Corresponding authors:

Zhu Liying, Email: zlyhappy@njtech.edu.cn, Tel: +86-25-58139430,

School of Chemistry and Molecular Engineering, Nanjing Tech University, Nanjing 210009, PR China.

Ling Jiang, Email: jiangling@njtech.edu.cn, Tel: +86-25-58139430,

College of Food Science and Light Industry, State Key Laboratory of Materials-Oriented Chemical Engineering, Nanjing Tech University, Nanjing 211816, China.

1. Pictures and Tables Section

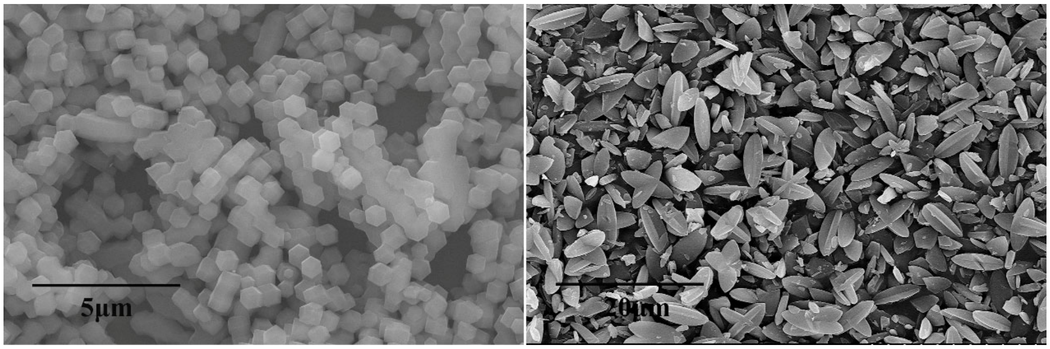

Figure S1. The SEM image of D- ZIF-8 (**left**) and P-ZIF-8 (**right**).

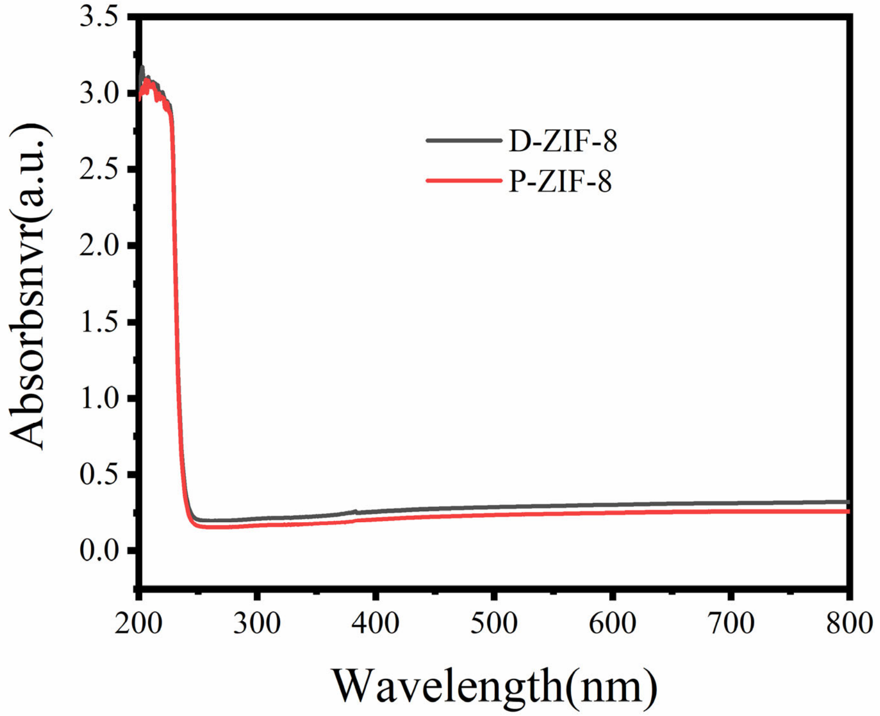

Figure S2. The UV full-wavelength scan of the two form of ZIF-8.

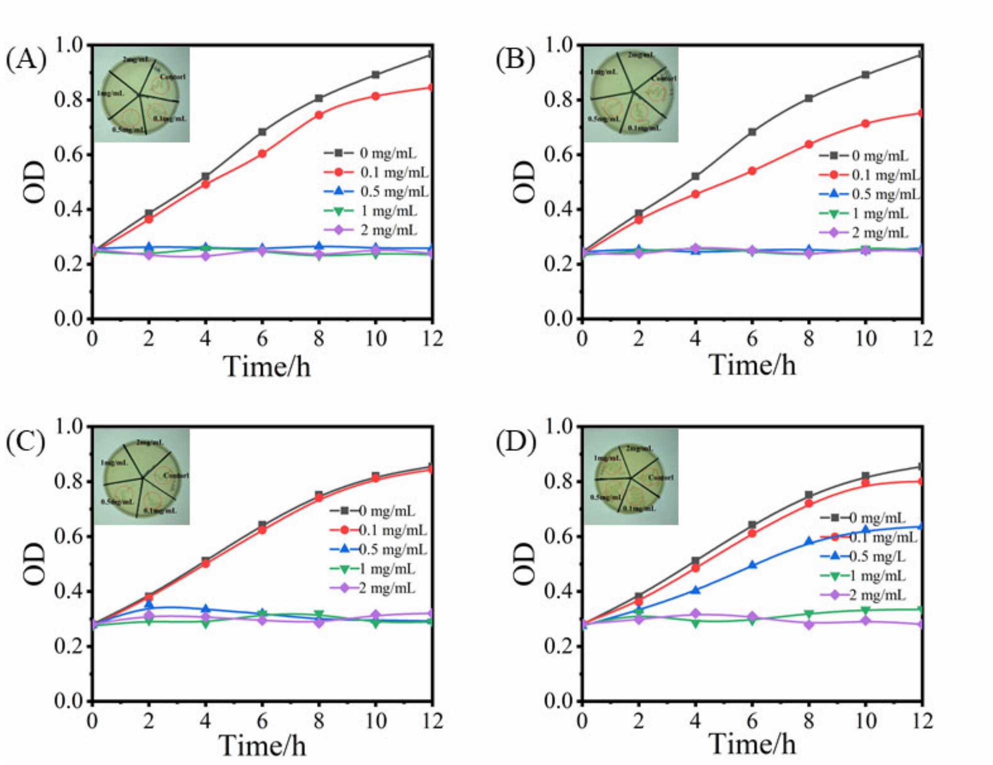

Figure S3. Antibacterial curve and solid plate diagram of the ZIF-8. (**A**)P-ZIF-8+*E. coli*; (**B**) D-ZIF-8+*E. coli*; (**C**) P-ZIF-8+ *S. aureus*; (**D**) D-ZIF-8+ *S. aureus*.

Table S1. MIC and MBC of ZIF-8

Materials	MIC(*E. coli*)	MBC(*E. coli*)	MIC(*S. aureus*)	MBC(*S. aureus*)
P ZIF-8	0.5 mg/ml	1 mg/ml	0.5 mg/mL	1 mg/mL
D ZIF-8	0.5 mg/ml	1 mg/ml	1 mg/mL	2 mg/mL

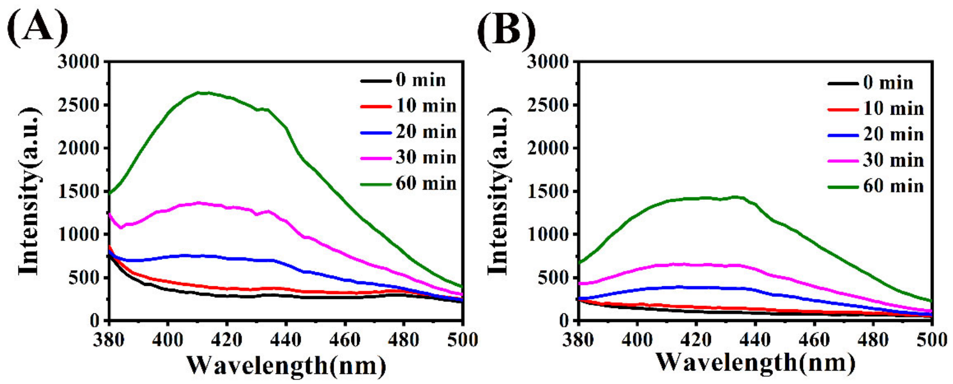

Figure S4. Fluorescence intensity of TA excited by ZIF-8 under illumination. (**A**) D-ZIF-8; (**B**) P-ZIF-8.

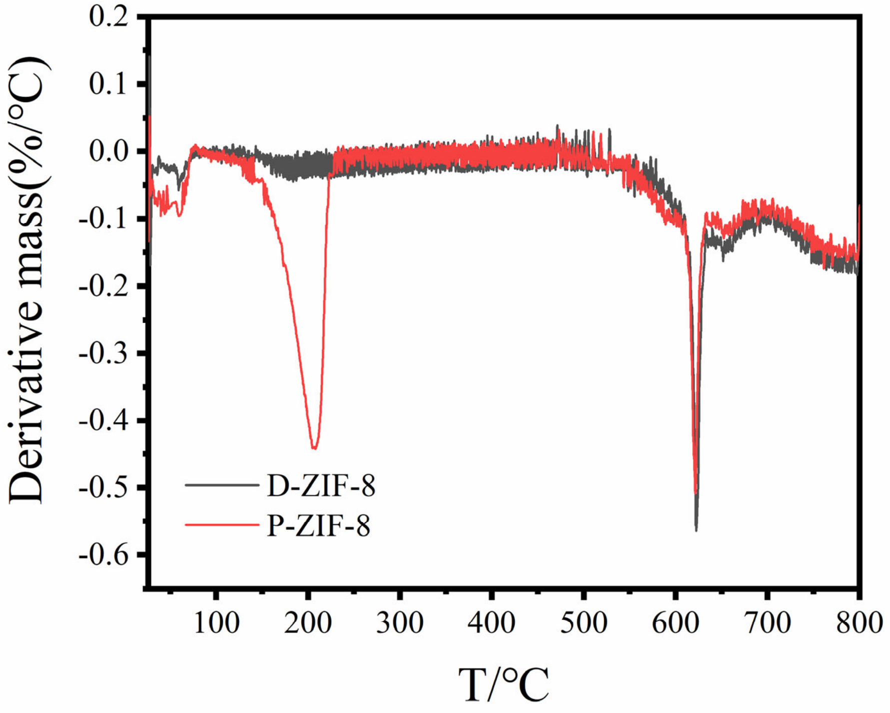

Figure S5. DTG of D-ZIF-8 and P-ZIF-8.

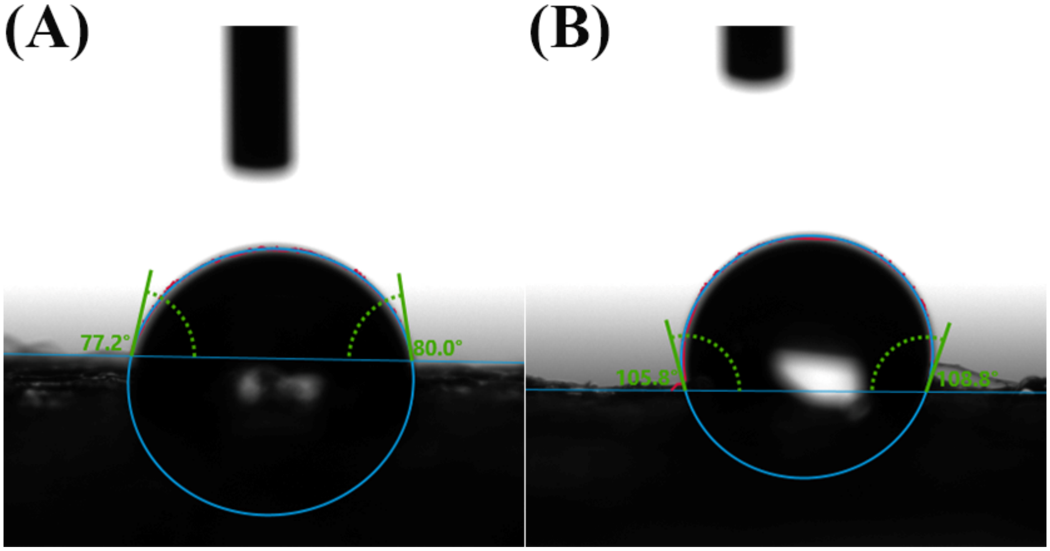

Figure S6. Contact angle with 20 μL water. (**A**) Fiber; (**B**) ZIF-8-Film.

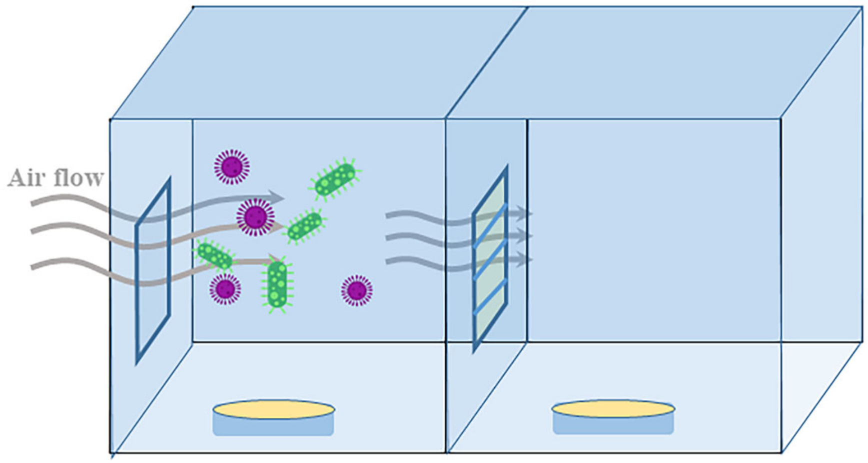

Figure S7. Schematic diagram of filter test device.

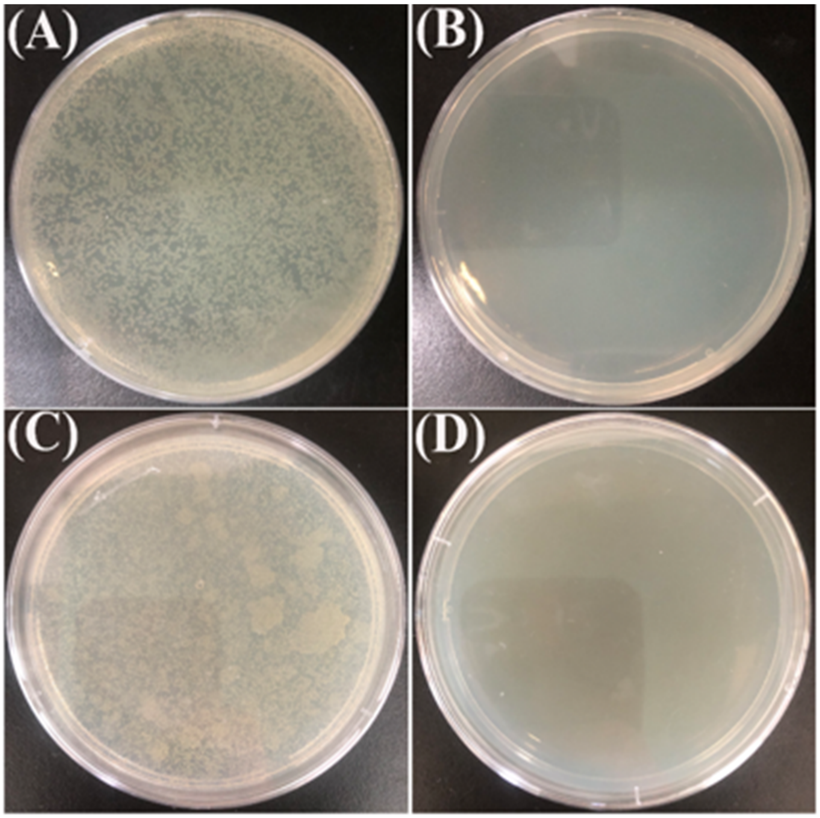

Figure S8. (**A**) A solid medium in an environment fill with *E. coli*; (**B**) A solid medium in an environment fill with *E. coli* isolated with ZIF-8-Film; (**C**) A solid medium in an environment fill with *S. aureus*; (**D**) A solid medium in an environment fill with *S. aureus* isolated with ZIF-8-Film.

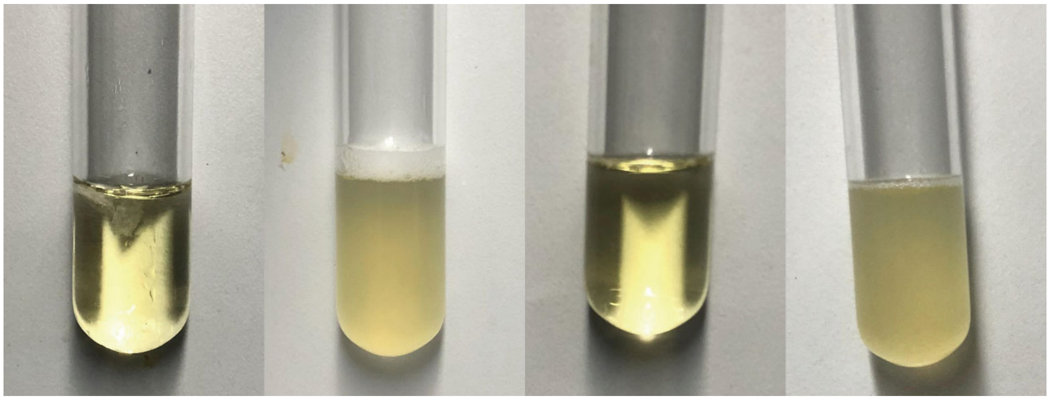

Figure S9. From left to right are ZIF-8-Film+*E. coli*, fiber+*E. coli*, ZIF-8-Film+*S. aureus*, fiber+*S. aureus*.

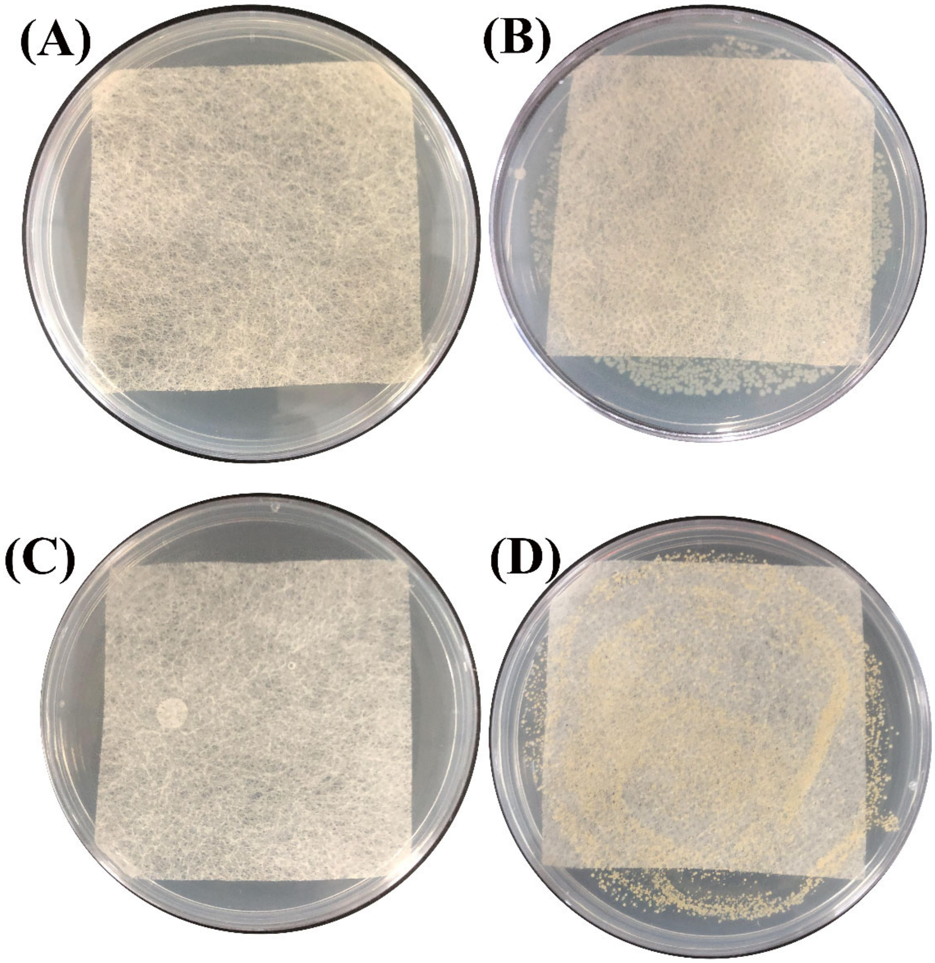

Figure S10. (**A**) ZIF-8-Film+*E. coli*;(**B**) fiber+*E. coli*;(**C**) ZIF-8-Film+*S. aureus;*(**D**) fiber+*S. aureus.*
